# Recruiting general practitioners and patients with dementia into a cluster randomised controlled trial: strategies, barriers and facilitators

**DOI:** 10.1186/s12874-021-01253-6

**Published:** 2021-03-30

**Authors:** Sonia Lech, Julie L. O’Sullivan, Leonard Wellmann, Juliana Supplieth, Susanne Döpfmer, Paul Gellert, Adelheid Kuhlmey, Johanna Nordheim

**Affiliations:** 1 Charité – Universitätsmedizin Berlin, corporate member of Freie Universität Berlin, Humboldt-Universität zu Berlin, and Berlin Institute of Health, Institute of Medical Sociology and Rehabilitation Science, Charitéplatz 1, 10117 Berlin, Germany; 2 Charité – Universitätsmedizin Berlin, corporate member of Freie Universität Berlin, Humboldt-Universität zu Berlin, and Berlin Institute of Health, Institute of General Practice, Berlin, Germany

**Keywords:** Primary care, Recruitment, Cluster randomised controlled trial, Dementia

## Abstract

**Background:**

Recruitment of general practitioners (GPs) and their patients is reported as one of the most challenging steps when undertaking primary care research. The present paper describes the recruitment process of a cluster randomised controlled trial (cRCT) aiming to improve dementia care in the primary care setting.

**Methods:**

Recruitment data was analysed descriptively using frequency tables to investigate comparisons of recruitment rates and results of different recruitment strategies as well as reasons for participation and non-participation of GPs, patients with dementia (PwD) and their caregivers.

**Results:**

Over a period of 23 months, *N* = 28 GPs were successfully included in the cRCT. This represents an overall recruitment rate of 4.6%. The most efficient strategy in terms of high response and low labour-intensity involved the dissemination of calls for participation in a GP research network. Most frequently reported reasons for GP’s participation were *Improvement of patient’s well-being* (*n* = 22, 79%) followed by *Interest in dementia research* (*n* = 18, 64%). The most common reasons for non-participation were *Lack of time* (*n* = 71, 34%) followed by *Not interested in participation* (*n* = 63, 30%). On a patient level, *N* = 102 PwD were successfully recruited. On average, each GP referred about *n* = 7 PwD (range: 1–17; *mdn* = 6; IQR = 3.5) and successfully recruited about *n* = 4 PwD (range: 1–11; *mdn* = 3; IQR = 3.5).

**Conclusion:**

First, our findings propose GP research networks as a promising strategy to promote recruitment and participation of GPs and their patients in research. Second, present findings highlight the importance of including GPs and their interests in specific research topics in early stages of research in order to ensure a successful recruitment. Finally, results do not support cold calls as a successful strategy in the recruitment of GPs.

**Trial registration:**

The trial was prospectively registered with the ISRCTN registry (Trial registration number: ISRCTN15854413). Registered 01 April 2019.

## Background

General practitioners (GPs) play a paramount role in dementia care [1–6]. They are often the first point of contact for patients with dementia (PwD) and play a key part in both diagnosis [7–9] and management of the disease [1, 10–12]. Despite the central role of GPs in the care of dementia, primary care-based interventions to assist GPs and PwD remain rare. The involvement of GPs in research to improve dementia care remain crucial. However, the recruitment of GPs in health research poses a major obstacle and barriers of recruitment have been reported in various areas of health research [13–18]. Barriers to GP’s recruitment and research participation were found to be manifold, including lack of time [13, 16, 17, 19, 20] and administrative burden [20, 21]. Further, in a systematic review poor communication by trial coordinators, difficulties of understanding research methods, concerns about possible harms for patients and feelings of being overwhelmed by too many research requests without being addressed as a real research partner were identified as barriers [22]. In Germany, despite an increasing awareness of the need for clinical trials in primary care there is no long tradition of involving GPs in research, and clinical trials in primary care are still under-represented [23]. In other countries, this tradition has existed significantly longer, as for example in the Netherlands, UK and the US [21, 23–25].

When it comes to primary care research, not only the recruitment of GPs proposes an obstacle, but also the recruitment of patients [22, 26–28]. For example, in the United Kingdom, less than one third of health studies in primary care reach their target number of patients, partly due to the overestimation of recruitable patients by GPs [27]. This frequently occurring case, also known as “*Lasagna’s law*” [29], inevitably leads to challenges [30]. Despite necessary long-term commitments of GPs, the number of patients actually available for recruitment turns out to be many times lower than initially estimated. Recruitment of patients into randomised controlled trials (RCT) was proven to be particularly challenging [31]. RCTs require a sufficiently large number of participants and failure to reach patient recruitment targets often lead to insufficient statistical power or discontinuation of trials [30, 32]. Reasons for difficulties in the recruitment of patients within the primary care setting are manifold. For example, in a qualitative study investigating perceived barriers among GPs towards recruiting patients into RCTs lack of confidence in introducing research participation requests to their patients was found as one main reason [19]. Data protection regulations also make it particularly difficult to contact patients directly [33]. Particularly in studies with a limited funding period, extending periods of recruitment represent a major problem [34].

To sum up, recruitment of GPs and their patients is considered as one of the most challenging steps in health research and, although this difficulty has long been recognised as such, there is a lack of effective strategies to overcome it [14, 18, 31]. To date there is no comprehensive publication on the recruitment methods and facilitating and/or inhibiting factors in the recruitment of GPs and their patients with dementia into a RCT.

### Aim of study

The present study aims to describe the recruitment process and the results of the recruitment of GPs recruited within the DemTab trial. The main focus of the present paper lies on the recruitment of GPs. In addition, the results of the recruitment of PwD and their caregivers are presented. The objective is to reflect on efforts and risks of different recruitment strategies applied in the present study. Furthermore, we investigate factors that have facilitated or hampered recruitment will be examined. We aim to contribute to a better understanding of barriers and facilitators of the recruitment of GPs and their patients.

## Methods

### Study design

To examine our research questions, data was used from the DemTab study, a two-arm cluster randomised controlled trial (cRCT) with the objective of the development and evaluation of a tablet-based intervention aiming to improve primary care for PwD and their caregivers^1^ in Berlin and surrounding area. A study protocol of the DemTab study was published elsewhere [35]. The study was conducted and reported in accordance with the CONSORT guidelines for cRCT and ethical approval was obtained by the ethics committee of the Charité – Universitätsmedizin Berlin (EA1/085/19). The trial was prospectively registered with the ISRCTN registry (Trial registration number: ISRCTN15854413).

In the first part of the DemTab study a feasibility study was conducted. In order to collect perspectives and needs regarding the treatment of dementia in primary care and include these in the development of the intervention, interviews and a workshop with GPs and other important actors from the ambulatory care setting were carried out. A publication on the feasibility study is currently underway. Following the feasibility study, the intervention was developed and implemented.

### Intervention

The tablet-based intervention is composed of multiple functions and applications. The main functions include, for example, a checklist, similar to a conversation-guide which supports GPs in guideline-based care. Another function enables GPs to communicate via messages with PwD and their caregivers. GPs received each a tablet and PwD and their caregiver shared a separate one. Participants of the intervention group were provided (if necessary) with internet access. A training on the tablet-based intervention was conducted prior to the intervention’s beginning to ensure participation, followed by a nine-month tablet-based intervention with the aim to improve guideline-based dementia care. Participants of the control group receive standard healthcare by their GPs and additionally an information handbook on dementia at the beginning of the trial. All participant are encouraged to use the tablet as often as desired – the usage of the tablet is voluntary and there are no further commitments in terms of frequency or quality of usage. The trial is currently ongoing. A more detailed description of study design, sampling methods, variables and procedures can be found in Lech et al. [35].

### Participants and procedure

The recruitment process was comprised of two stages: first, GPs were recruited followed by the recruitment of PwD (and their caregivers). The original recruitment target of *N* = 20 for GPs and *N* = 202 for PwD and their caregivers was based on a sample size calculation using GPs ratings and proxy ratings of caregivers from medical record information as primary outcome [36]. Due to challenges in the recruitment of GPs and PwD the primarily estimated sample size could not be reached. Consequently, literature was reviewed de novo [36, 37]. When in 2017 a comparable cRCT from Germany evaluated a guideline-oriented intervention (Dementia Management Program) for PwD in primary care using a patient-related primary endpoint, a new power calculation at patient level was conducted based on the reported medium-sized effect of Cohen’s d = 0.5 [37]. Based on that study, a new power calculation using the software G*Power 3.1 yielded an estimated new total sample size of *N* = 102 or *n* = 52 per group at a type I error rate of alpha = 0.05 and a statistical power of 1-β = 0.8. These calculations take into account the variance between GPs (ICC = 0.03) and a drop-out rate of 18% at follow-up, as found by Vickrey et al. [36].

Inclusion criteria for GPs were defined as (1) currently operating as GP, (2) meeting technical requirements (internet connection), (3) willing to participate in a training, and (4) signed cooperation agreement. Exclusion criteria for GPs were a planned absence or closing of the practice for longer than 4 weeks during the study period. Further, GPs with a lack of PwD currently treated in practice were also not included. Inclusion criteria for PwD were defined as (1) diagnosis of dementia obtained prior to the beginning of the trial (acc. to ICD-10, F00-F03, G30, G31.0 and G31.82), (2) living at home (outpatient care), (3) availability of a caregiver, and (4) signed informed consent (if they are still legally authorised to sign, otherwise through a person holding the power of attorney). Exclusion criteria for PwD were (1) other mental and behavioural disorders (acc. to ICD-10, F10–29, except for F10.1, F17.1 or F17.2, as well as F32.2 and F32.3), (2) a planned hospital or rehabilitation stay longer than 4 weeks, and (3) a planned relocation to an inpatient care-facility or nursing home within the study period. Inclusion criteria for caregivers were defined as (1) living with or regularly visiting PwD and (2) signed informed consent. Exclusion criteria included a planned absence longer than 8 weeks during the study period.

Assessments of primary and secondary outcomes were conducted before the intervention (baseline) and after the intervention (post intervention) in both groups. Primary outcome is defined as adherence to dementia guideline recommendations after 9 months. Secondary outcomes include various health outcomes assessed in PwD (e.g., quality of life) and caregivers (e.g., caregiver burden). Randomisation was conducted at a GP level to avoid contamination across groups (cluster randomisation). At the end of the study, participating GPs from both treatment groups were to receive a financial compensation of 100 EUR for each PwD successfully recruited. Furthermore, all GPs were to receive a tablet computer permanently. Participating PwD and caregivers did not receive any direct financial compensation, though all study participants enter a lucky draw and receive the opportunity of winning a tablet.

### Recruitment of GPs, PwD and their caregivers

Overall, in line with prior research and the Dillman’s Total Design Approach [38, 39], recruitment strategies for GPs included personalised invitations and letters, comprehensive information material on the study rationale, goals and design, follow-up calls and endorsement from the research team via telephone, reply paid envelopes as well as a financial compensation in case of participation. In the present study, the recruitment of GPs was conducted in *three recruitment rounds*. The first and the second recruitment round were intended prior to the beginning of recruitment. The third recruitment round was added during the ongoing recruitment process to ensure the necessary sample size. Partially, recruitment of all rounds took place simultaneously. In the *first recruitment round*, calls for participation and advertisements of the DemTab study were published in a variety of general practice related publications and newsletters through different networks. A main strategy was the dissemination of a call for participation in a regular newsletter of a research network of general practitioners in and around Berlin established by the Institute of General Practice of the Charité – Universitätsmedizin Berlin. Further, a total of three advertisements in general practice related publications was disseminated and four ads were published on Facebook pages related to dementia. The project and call for participation were presented at two trainings for GPs in Berlin. Further, advertisements through further GP networks (e.g. presentation of the DemTab study in quality circles of primary care) lead to recommendations and referrals of potentially interested GPs (snowball sampling). In the *second recruitment round*, a sample of GPs (*n* = 486) was randomly selected from a database of the Statutory Health Insurance Physicians in Berlin (KV Berlin). At first, GPs received personalised letters with comprehensive information material about the DemTab study followed by a phone call. The low initial response (none of the contacted GPs got back to the research team based on letters) resulted in directly contacting GPs via phone (cold calls), instead of sending out letters first. Additionally, rural areas in the vicinity of Berlin were included and contacted via cold calls. Finally, a *third recruitment round* included face-to-face recruitment of GPs in *n* = 116 general practices in Berlin. Practices were selected primarily on the basis of the official number of older people living in the district, starting with the districts with the highest numbers. Practices were visited between October 2019 and February 2020. An in-person meeting with GPs was intended and a package of information material on the DemTab study was distributed directly to GPs in their practice.

### Inclusion of GPs and PwD

Generally, once GPs showed interest in participating, a cooperation agreement accompanied by a reply-paid envelope was provided. A signed cooperation agreement was considered as a successful inclusion. Further, included GPs filled out a baseline survey. In a second step, GPs were required to recruit PwD in their practice. For this purpose, GPs were provided with information material and leaflets in order to ensure a successful recruitment of their PwD. Once GPs obtained permission from PwD and/or caregivers, patient’s contact details were shared with the research team. The research team then contacted PwD and/or caregivers via phone in order to provide a detailed description of the study for each participant. Once PwD and/or caregivers indicated interest in the participation over the phone, detailed study information and an informed consent form, accompanied by a reply-paid envelope was sent to their homes. A signed informed consent was considered as a successful inclusion. Further, included PwD and their caregivers filled out a baseline survey.

### Data analysis

Data on the recruitment of GPs and PwD was collected and documented by the research team. Baseline data was obtained from all successfully recruited participants. Documentation of the recruitment process of GPs includes data on (1) number of contacted GPs, (2) amount of successfully recruited GPs and (3) drop-out rates for each recruitment round. Further, recruitment rates (number of successfully participating GPs divided by the number of GPs contacted for recruitment) and recruitment ratios (number of successfully participating GPs in relation to the final GP sample) for each recruitment round were calculated. Data on reasons for participations was analysed based on a survey filled out by each successfully recruited GP (Item: “*Why did you choose to participate in this research study?*”, response categories: “*Improvement of patient’s wellbeing”, “Interest in dementia research”, “Improvement of patient’s health”, “General interest in research”, “Better insights in new health technologies”, “Assistance in patient management”, “Assistance in dementia care”, “Expense allowance”* and “*Other reasons”,* multiple responses possible). Data on reasons for non-participation was collected from each GP who was successfully contacted but declined participation (Question: “Why did you choose not to participate in this research study?”). Responses provided were documented and coded (“*Lack of time”, “Not interested in participation”, “Not interested in research in general”,*” *Did not see any added value in participation”,* and “*Other reasons”,* multiple responses possible*).*

Documentation of the recruitment of PwD includes data on (1) number contacts of PwD provided by GPs, (2) number of successfully recruited PwD within each GP practice and (3) data on drop-out rates. Further, recruitment rates (number of successfully participating PwD divided by the number of PwD contacted for recruitment) were calculated. Data on reasons for participations was analysed based on a survey filled out by each successfully recruited PwD and their caregiver (Item: “*Why did you choose to participate in this research study?*”, response categories: “*Improvement of patient’s wellbeing”, “Interest in dementia research”, “Improvement of patient’s health”, “Improvement of communication with GP”, “Improvement of disease management”, “Assistance and discharge due to technology”, “Better insights in new health technologies”,” Participation in a raffle of a Tablet computer”* and “*Other reasons”,* multiple responses possible). Data on reasons for non-participation was collected via phone from each PwD/and or caregiver who declined participation (Question: “*Why did you choose not to participate in this research study?*”). Responses provided were documented and coded *(“High care burden”, “Health reasons/advanced dementia/age”, “Not interested in participation”, “No further explanation”, “Technology-related rejection”, “No need for intervention”* and *“Other reasons”,* multiple responses possible). Data was analysed descriptively using frequency tables to explore comparisons of recruitment rates and recruitment ratios as well as results of recruitment strategies. For the descriptive analysis SPSS version 25 was used. Efforts of different recruitment strategies was ranked based on researchers experience and perception.

## Results

### Recruitment of GPs

**Fig. 1:**
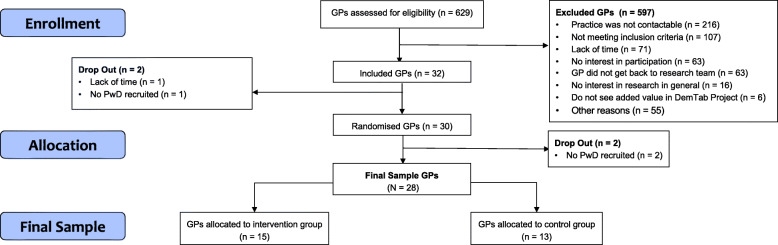
Flowchart of the recruitment of GPs

The recruitment of GPs was undertaken between June 2018 and March 2020 in the region of Berlin, Germany and surrounding areas. First, a total of *n* = 32 GPs was recruited (i.e., signed a cooperation agreement) into the study. However, due to early drop out during the recruitment phase, the final GP sample consisted of *N* = 28 GPs who successfully participated in the study. The recruitment process is summarised in Fig. 1. Results of all recruitment rounds separately are described in depth below.

#### First recruitment round

In the first recruitment round, all efforts resulted in *n* = 11 interested GPs contacting the research team. Out of these *n* = 11 GPs who contacted us, a total of *n* = 7 GPs was successfully recruited into the study. This proposes a recruitment rate of almost 64%. Out of *n* = 11 who contacted us, more than a half of GPs (*n* = 6) contacted us based on the newsletter of the GP research network. Out of these *n* = 6 GPs a total of *n* = 5 GPs were successfully recruited into the study (83%). Further, *n* = 6 GPs were contacted through snowball sampling, only *n* = 1 was successfully recruited (17%). To sum up, from a total of *n* = 17 eligible GPs in the first recruitment round, *n* = 8 GPs were successfully recruited into the study. However, *n* = 1 was not able to recruit any PwD and therefore was coded as a drop out after randomisation. This results in *n* = 7 GPs successfully participating in the study. Overall, the recruitment rate for the first recruitment round accounts for approximately 41% (*n* = 7). GPs recruited in this round represent 25% (*n* = 7) of the final GP sample.

#### Second recruitment round

In the second recruitment round *n* = 486 GPs (out of a total of N ≈ 2000 GPs) from all of the 23 districts in Berlin were randomly drawn from a database (KV Berlin). The first *n* = 276 GPs were contacted via mail and phone, followed by *n* = 210 GPs who were contacted only via phone. Out of all GPs contacted in this round (*n* = 486), only *n* = 271 were successfully reached. In total, this strategy resulted in *n* = 18 GPs included in the study. However, *n* = 3 (*n* = 2 before randomisation, *n* = 1 after randomisation) GPs dropped out leading to *n* = 15 successfully participating GPs. This proposes a recruitment rate of about 3% (*n* = 15). GPs recruited in this round represent 54% (*n* = 15) of the total GP sample.

#### Third recruitment round

**Fig. 2:**
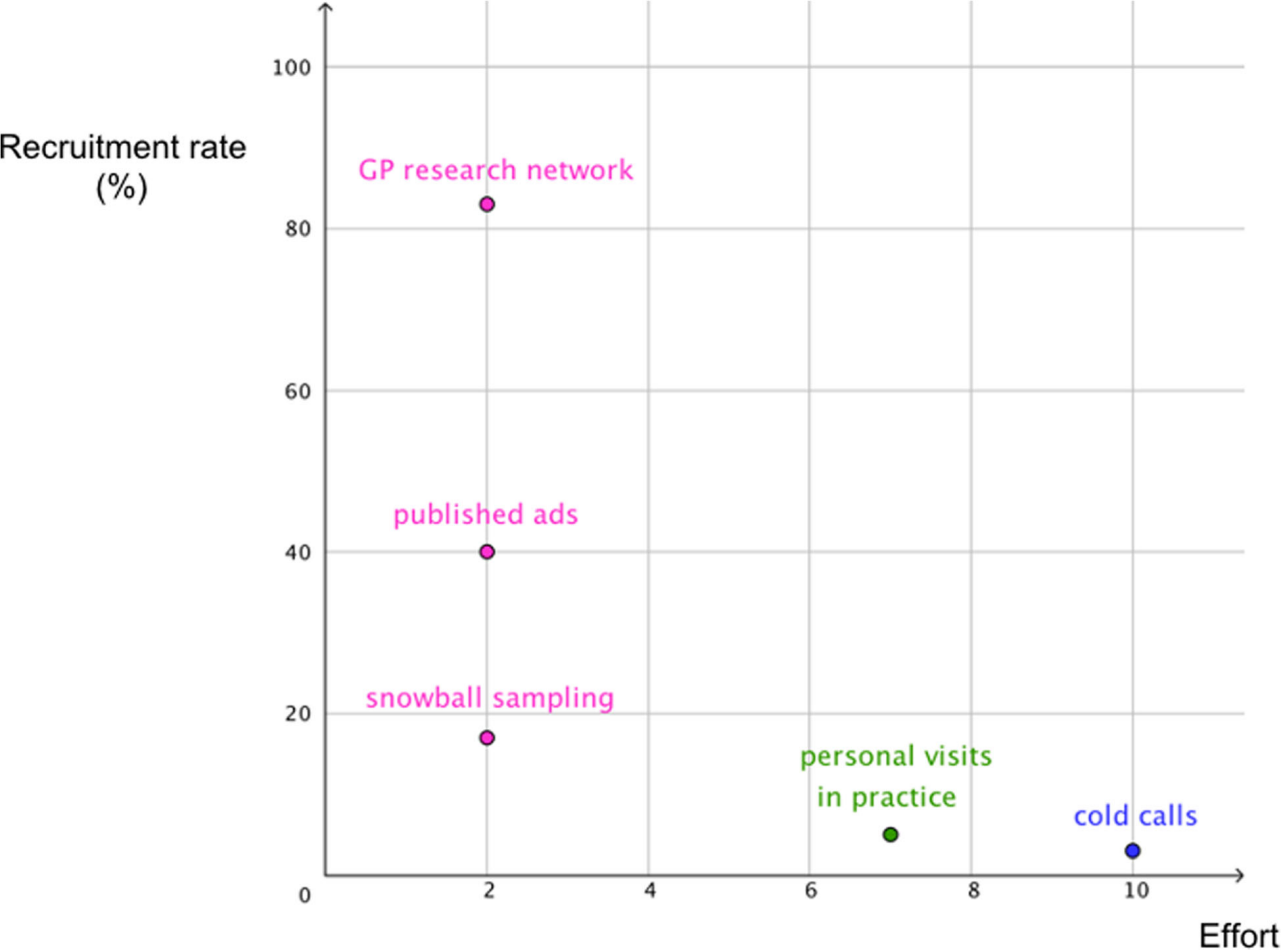
Estimated efficiency of recruitment strategies and rounds for GPs

In the third recruitment round GPs were visited directly in their practice. Based on the highest proportion of elderlies per district, GPs from nine of the 23 districts of the city Berlin were randomly chosen and *n* = 116 practices were visited on site. Out of all GPs visited in this round (*n* = 116), only *n* = 80 were successfully reached. A total of *n* = 6 GPs successfully recruited in this recruitment round. This proposes a recruitment rate of 5% (*n* = 6). GPs recruited in this round represent about 21% (*n* = 6) of the final GP sample. An overview of all recruitment rates and recruitment ratios can be obtained from Table 1. Figure 2 aims to visualise the efficiency (proportion of recruitment rate and effort of recruitment) of the different recruitment strategies and rounds of the present study.

**Table 1:** Overview of recruitment rates and recruitment ratios per round of GPs

GP Recruitment round	Contaced GPs (N)	GPs recruited (N)^**a**^	Drop Out (N)	Recruitment rate (%)^**b**^	Recruitment ratio (%)^**c**^
First recruitment round	17	8	1	41.2	25.0
Second recruitment round	486	18	3	3.1	53.6
Third recruitment round	116	6	0	5.2	21.4

*Note.* ^a^ includes GPs who signed informed consent. ^b^ Recruitment rate was calculated as number of successfully participating GPs (recruited GPs minus drop-outs) divided by the number of GPs contacted for recruitment. ^c^ Recruitment ratio was calculated based on the ratio of successfully participating GPs (recruited GPs minus drop-outs) and the final GP sample (*N* = 28)

#### Reasons given for participation and non-participation of GPs

The most commonly mentioned reason for participation was *Improvement of patient’s well-being* (*n* = 22, 79%) followed by *Interest in dementia research* (*n* = 18, 64%). Further, across all recruitment rounds, *N* = 107 GPs (34%) were successfully reached but did not meet inclusion criteria. The most frequent inclusion criteria not met was because of *Few eligible PwD* (*n* = 40, 37%) and certain *Disease specialisations of the practice* (e.g. on diabetes) (*n* = 22, 21%). In a total of *n* = 211 cases GPs were successfully reached but denied participation. Out of *n* = 211 GPs who denied participation, reasons for non-participation were inquired via phone and documented. The most common reason for non-participation was *Lack of time* (*n* = 71, 34%), followed by *Not interested in participation* (*n* = 63, 30%). Reasons for participation and non-participation given by GPs can be obtained from Table 2.

**Table 2:** Summary of reasons for participation and non-participation provided by GPs

	Number of GPs	Proportion %
**Reasons for participation**^**a**^		
Improvement of patient’s wellbeing	22	78.6
Interest in dementia research	18	64.3
Improvement of patient’s health	17	60.7
General interest in research	15	53.6
Better insights in new health technologies	12	42.9
Assistance in patient management	12	42.9
Assistance in dementia care	12	42.9
Expense allowance	2	7.4
Other reasons	8	28.6
**Reasons for non-participation**^**b**^		
Lack of time	71	33.7
Not interested in participation	63	29.9
Not interested in research in general	16	7.6
Did not see any added value in participation	6	2.8
Other reasons	55	26.1

*Note.* ^a^ *N* = 28 GPs, ^b^ *n* = 211 GPs

### Recruitment of PwD and their caregivers

**Fig. 3:**
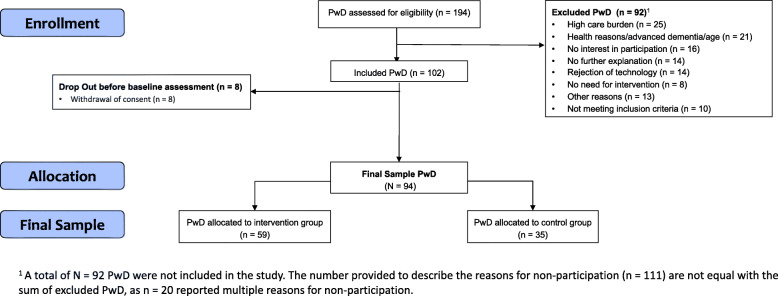
Flowchart of the recruitment of PwD

The recruitment of PwD and their caregivers was conducted between May 2019 and July 2020. A total of *n* = 194 contact details of PwD were provided by all *N* = 28 GPs. Figure 3 shows a flow chart of recruitment of PwD.

A total of *N* = 102 PwD were successfully recruited into the study. Overview of the recruitment descriptive statistics can be obtained from Table 3. On average, each GP referred about *n* = 7 PwD (range: 1–17; *mdn* = 6; IQR = 3.5), out of which on average about *n* = 4 PwD (range: 1–11; *mdn* = 3; IQR = 3.5) were successfully recruited. The overall recruitment rate for PwD was 54%.

**Table 3:** Overview of descriptive statistics of the recruitment of PwD

	Patients contacts provided by GPs					Patients successfully recruited into the study				
	n	Range	Mean (SD)	Median	IQR	n	Range	Mean (SD)	Median	IQR
**Total**	194	1–17	6.79 (3.91)	6.0	3.5	102	1–11	3.64 (2.53)	3.0	3.5
**Intervention**	124	2–17	8.13 (4.70)	7.0	8.0	67	1–11	4.47 (3.11)	3.0	5.0
**Control**	70	1–8	5.23 (1.92)	5.0	3.0	35	1–4	2.69 (1.11)	3.0	2.0

*Note. N* = 102 PwD. *SD* Standard Deviation, *IQR* Interquartile range

#### Reasons given for participation and non-participation by PwD and/or caregivers

The most commonly mentioned reason for participation was *Improvement of patient’s well-being* (*n* = 73, 82%) followed by *Interest in dementia research* (*n* = 69, 78%). Out of *n* = 194 PwD contact information provided by GPs, *n* = 9 PwD did not meet the inclusion criteria. Further, *n* = 82 PwD denied participation. Reasons for non-participation were inquired from PwD and/or caregivers and documented. The most common reason for non-participation provided was *High care burden (n* = 25, 22%) followed by *Health reasons/advanced dementia/age* (*n* = 21, 19%). Reasons for participation and non-participation given by caregivers of PwD can be obtained from Table 4.

**Table 4:** Summary of reasons for participation and non-participation provided by caregivers

	Number of caregivers	Proportion %
**Reason for participation**^**a**^		
Improvement of patient’s wellbeing	73	82.0
Interest in dementia research	69	77.5
Improvement of patient’s health	65	73.0
Improvement of communication with GP	58	65.1
Improvement of disease management	41	46.1
Assistance and discharge due to technology	35	39.3
Better insights in new health technologies	31	34.8
Participation in a raffle of a tablet computer	16	18.0
Other reasons	13	14.6
**Reason for non-participation**^**b**^		
High care burden	25	22.5
Health reasons/advanced dementia/age	21	18.9
No interest in participation	16	14.4
No further explanation	14	12.6
Technology-related rejection	14	12.6
No need for intervention	8	7.2
Other reasons	13	11.7

*Note.* ^a^ *n* = 89 caregivers, ^b^ *n* = 82 PwD and/or caregivers

## Discussion

Recruitment of GPs and their patients is reported as one of the most difficult tasks in the implementation of primary care research. The key objective of the present paper was to describe the recruitment process and provide result of the enrolment of GPs and their PwD of a cRCT aiming to examine the effect of a tablet-based intervention. Recruitment was organised in two parts: first GPs were recruited, followed by the recruitment of PwD and their caregivers within each cluster.

### Recruitment of GPs

Of all GPs who were eligible for participation almost 5 % responded to take part in the study, which is comparable to previous research [40, 41]. For example, Williamson et al. [42] reported an initial overall response rate of 4.1% in their study. Further, the original target of recruiting *n* = 20 GPs was accomplished. However, similarly to other studies, recruiting time and resources had to be extended [34, 43]. A variety of recruitment rounds and strategies were applied in order to maximise successful recruitment. Recruitment efforts and success rates differed across strategies. The most efficient strategy in terms of absolute numbers was the second recruitment round. However, this strategy was proven to be extremely labour-intensive as it included cold calls of GPs in their practice. Initially, it was planned to send out information material and leaflets with calls for participation via mail to each practice. However, none of the GPs that were contacted via mail ever responded. This finding has been already reported in previous research [40, 44]. Thus, follow-up calls were initiated and indicated a better response which is why a decision was made to forgo contacting GPs via mail and directly contact them via phone. This experience is in line with previous research. For example, Parkinson et al. [40] found in a sample of non-responding GPs that the vast majority had not seen the invitation which was sent via mail, suggesting it had not been passed on by administrative staff. Despite it being more fruitful, cold calls lead to new challenges. First, most GPs in Germany are only available during patient consultation hours. Consequently, a variety of GPs were occupied and therefore often not reachable. Second, once a primary care practice was successfully reached, the phone was answered almost exclusively by non-GP staff (e.g., receptionists, doctor’s assistance or practice nurses). The present experience has shown that many times non-GP staff were occupied with daily work and due to practice structures not able and/or interested in passing on study information or requests for recalls to GPs. Further, engaging and rapport building with non-GP staff emerged as difficult. This hurdle is in line with previous work examining the role of non-GP staff in recruitment processes [6, 40, 42, 45], acknowledging the increasingly busy work environment in general practices [17, 23, 45]. To sum up, in the present study cold-calling GPs was found to be challenging, ineffective, extremely labour-intensive and opposite of the collaborative structure of primary care, all observations in line with previous research. However, in terms of the external validity and generalisability of study results, cold calls enable a random and systematical recruitment of GPs. If possible, future research should assess the labour-intensity and costs individually for each study in order to plan and budget accordingly. As shown in Fig. 2, the most efficient strategy in terms of high response and low labour-intensity was proved to be the first round, especially the dissemination of calls for participation in a GP research network. More than half of GPs recruited in this recruitment round were recruited via the GP research network. This is in line with previous research and the current trend to establish national research network for GPs [23, 26, 40, 46]. Further, whereas the representativeness of GPs from research networks may be limited, patients of these practices are found to be representative [47]. In the present study, recruitment within a GP network was not only fruitful, but also did not require any financial and human resources. Based on the present finding and previous research, we strongly encourage the promotion and advertisement of GP networks. In Germany, the recent initiative (*Initiative of German Practice-Based Research Networks – DESAM-ForNet*; https://www.desam-fornet.de/ initiative-deutscher-forschungs praxennetze-desam-fornet/) aims to compose a wider research network by merging six regional research networks into one united German research network. GP networks might not only support with the recruitment of GPs into trials but represent potential for the provision of trainings for GPs who are interested in research methods and participation as well as recruitment of patients within their practice. In GP networks, GPs are seen as a research partner and not only as a provider of eligible patients. Their view on the relevance and feasibility of a research project at the planning stage of a project has the potential to improve the acceptability and thus participation of GPs in research [48–50]. With regard to snowball sampling, successfully recruited GPs of the present study were provided with additional recruitment material and asked to invite GP colleagues to participate in the study. This strategy has led to numerous referrals of potentially interested GPs, unfortunately only one was successfully included. However, previous research has recommended physician-to-physician recruiting as a promising recruitment strategy for primary care [51, 52]. Thus, future research may consider physician-to-physician recruitment. In terms of personal visits of GPs in their practice, based on present findings and previous research [51, 53, 54] we believe that well planned visits and a flexibility to individual practice styles may propose an effective recruitment strategy. In the present study, during our visit in GPs practices we provided GPs with a small package including information material on the study, flyers for patients, a required cooperation agreement in case of interest in participation as well as a reply-paid envelope. However, previous research has reported little or no effect of information leaflets and flyers on successful recruitment [53, 55].

Across all recruitment rounds, analysis of reported reasons of participation revealed that the *Improvement of patient’s well-being* as well as a certain *Interest in the research topic* were main reasons for participation. Findings that practitioner’s interest in the research topic facilitates recruitment is in line with previous studies [16, 56–58]. For example, a recent study conducted by Ferrand Devouge et al. [20] found that the relevance of a research topic for clinical practice was one main reason for participation. Our finding outlines the great importance of incorporating the role and views of GPs from an early stage on. However, the DemTab study was aiming to involve primary care perspectives from the onset of the study. Thus, interviews and a workshop with GPs were conducted prior to the intervention’s beginning in order to collect and analyse different perspectives and needs of dementia primary care and include these in the development of the study. However, the participation and involvement of GPs should commence at an earlier stage of research, namely in the development and design of research questions and project proposals. The present findings highlight the key role of GPs interest in certain research topic for their participation in research. In order to apply successful strategies for the recruitment of GPs that are congruent with the context of care delivery, it is highly instructive for future research to target active inclusion of GP’s views and needs in the early stage of research, ideally at the stage of development of research projects and proposal writing. The most common reason for non-participation was *Lack of time*. This finding is in line with previous research acknowledging time constraints and time-related difficulties for participation in primary care research [13, 16, 17, 19, 20]. In order for GPs to participate in research and development of new care models, structural barriers such as lack of time due to everyday business in general practices have to be.

### Recruitment of PwD

Recruitment of PwD emerged as challenging. Due to the cluster structure of the study and lack of PwD within one practice, recruitment of GPs had to be expanded. Even though we were able to recruit *N* = 28 GPs, only *N* = 194 contact details of PwD were forwarded by GPs. Consistent with previous research [22, 27, 59], in the present study the recruitment of patients into a cRCT through GPs has shown to be difficult and unsuccessful, as it did not result in the accomplishment of the primarily estimated sample size (*N* = 204). A variety of reasons can be drawn from the present study that may have contributed to a poor recruitment of PwD. First, at the beginning of the trial, most of GPs were too optimistic about eligible PwD in their practice. Later, GPs reported that it was much more challenging to recruit patients than expected. This phenomenon was already described by past research with the “*Lasagna’s law*” [29] and is in line with previous research [42, 60]. However, in the present study, on average, one GP referred seven PwD (*mdn* = 6). A study conducted by Page et al. [31] reported a median of two patients per GP recruited into a trial. Further, our experience indicates that time constraints at GP level may have contributed to poor recruitment. Despite of continuous follow-up calls and reminders, it was often pointed out, that GPs did not have time or forgot to recruit PwD. This observation is also in line with previous research [16, 30, 31, 61].

However, the present study examined a technology-based intervention for GPs, PwD and their caregivers. Despite of lack of interest in technology not being a main reason for non-participation in the present study, it may be that a technology-based approach for the improvement of care for the elderly population meets no particular interest. This assumption is in line with previous empirical work, examining attitudes and beliefs towards technology based (health) devices [28, 62] and may propose a reason for the poor recruitment of PwD and their caregivers. The main reason for non-participation of PwD and/or caregivers included *High care burden* followed by *Health reasons/advanced dementia/age*. Previous research has already acknowledged poor health status and old age as predictors for refusal of participation in health research [63–65]. For example, Jacomb et al. [66] found that cognitive impairment predicted refusal of research participation. Future research should operate towards the identification of effective strategies to overcome recruitment barriers of older patients and patients with dementia in order to successfully include these groups in research and public health approaches aiming to improve health care. In terms of reasons for participation given by caregivers of PwD the most common reasons mentioned were *Improvement of patient’s well-being*, followed by *Interest in dementia research*. Personal benefits have been already acknowledged as important drivers for participation in research [67, 68].

### Limitations

The results of the present study have to be considered in light of certain limitations. First of all, the study’s objective was the evaluation of a tablet-based intervention which limits the present results in terms of generalisability. Even though interest in and willingness to use technological based tools for the improvement of care are growing, practitioners are often found to be hesitant to new technologies [69–72]. Building on the present finding, that interest in a research topic plays a key role in the recruitment success of GPs, it may be that recruitment of GPs, PwD and their caregivers has proven complicated due to a technology-based intervention. For example, on a patient level, Foster et al. [28] found that a great proportion of patients rejected their participation in two linked randomised controlled telehealth trials because of a lack of ability to engage with telehealth or a lack of perceived need for it. However, in order to address and prevent structural and personal barriers of technology usage, GPs, PwD and their caregivers of the present study were provided with internet access, received a training and a handbook on the tablet usage prior to the beginning of the intervention. Further, analysis of reasons for non-participation did not reveal any major indication for technology related lack of interest in the study. Only 12.6% of PwD/and or caregivers declined participation due to technology related reasons. However, present findings have to be interpreted in the context of technology-based intervention studies. Especially, as the target population of the present study was elderly patient diagnosed with dementia. This limitation has to be taken into consideration when interpreting the present results. Second, an unbalanced sample size between control and intervention group has to be taken into account. In order to minimise the risk of recruiting a selective sample of patients, GPs were not informed about allocation for as long as possible. Due to the design and flow of study, as well as the challenges emerging during recruitment of PwD, in the course of the trial GPs were informed about their allocation, in some cases during the ongoing recruitment process of PwD. In order to avoid bias, GPs were asked to not inform PwD about the allocation of the practice. Further, the study nurse was blinded until after the baseline assessment. However, the average number of referrals slightly differed between groups: in the intervention group GPs referred on average *n* = 8 PwD, whereas in the control group *n* = 5 PwD were referred on average. Although we did not see large differences across intervention arms in recruitment rates (median of successfully recruited PwD equals three for both groups), there might be a risk of bias in terms that it may have been easier for GPs to motivate and recruit PwD into the intervention group.

### Practical implications

Based on the present findings, the following recommendations for the recruitment of GPs and their patients in primary care research in Germany can be drawn:

- *Cold calls remain labour-intensive and due to structural barriers in every day primary care practice unsuccessful, particularly for research projects dealing with low human and financial resources*
- *Primary Care Research Networks represent a valuable contribution to primary care research:*
  - ◦ Establishment of GP research networks
  - ◦ Trainings for GPs on research, participation and successful recruitment of patients
  - ◦ Dissemination of research projects and calls for participation among research interested GPs
- *The key role of research topics and their practical relevance for GPs*
  - ◦ Involvement of GPs in the research process from the early stage on:
    - ▪ Participation in the development of research questions
    - ▪ Participation in the writing of research proposals

## Conclusions

Barriers to GP recruitment identified in the present study were similar to those reported in previous research. To optimise recruitment of GPs in RCTs, research networks of GPs were found to be most efficient in terms of high response and low labour-intensity. Further, findings outline the great importance of involving GPs in early stage of research. Finally, results do not support cold calls as a successful strategy in the recruitment of GPs. Regarding recruitment of PwD and their caregivers, expectations of patient’s well-being improvement and interest in research topic were the most common reasons for participation.

## Data Availability

Data is stored in a non-publicly available repository. Data are however available from the corresponding author on request.

## Abbreviations

cRCT: Cluster randomised controlled trial
GP: General practitioner
ICC: Interclass correlation coefficient
IQR: Interquartile range
Mdn: Median
PwD: Patients with dementia
RCT: Randomised controlled trial
SD: Standard deviation
UK: United Kingdom
US: United States of America

## Footnotes

1 The target of the DemTab study included PwD who receive ambulatory care and their family or informal caregivers. However, in Germany dementia shared homes also count as ambulatory care. Therefore, we also included non-family caregivers from ambulatory care services such as dementia shared homes. We refer to family, non-family and caregivers as caregivers.
